# A New Feature

**Published:** 1917-01

**Authors:** 


### A New Feature.

Beginning with an early issue the JOURNAL will have a department devoted to brief exchanges of ideas and experiences. Every physician and surgeon has in his practices some unusual cases or experiences that are hardly of sufficient importance to justify a lengthy article, but which might, if briefly told, be of help to someone else. Or some physician may have met some difficult case about which he would like to have the opinion of others who have had similar cases, and may seek such information through this department. There is hardly a subscriber to the JOURNAL who is not in position to help a brother physician by relating something from his own practice. Most of them can take the time to write down a brief note of this kind, but would hardly undertake a more labored article.

Every reader of the JOURNAL is invited to become a contributor, making the reports as brief as possible and avoiding professional and technical terms where it can be done. Usually reports of this kind can be limited to two or three hundred words. Sit down and write something from your many experiences in which you think others might be interested.

